# Supplementary material for: Efficient open recruitment and perspectives of host families on medical student homestays in rural Japan
Source: PLoS One. 2022 Jan 26;17(1):e0263132. doi: 10.1371/journal.pone.0263132 (PMC8791476; doi:10.1371/journal.pone.0263132)
Supplement: Attachment — Submitted filename: Point-by-point_response_to_reviewer_comments_Ver.3.docx [file pone.0263132.s002.docx]

**Point-by-point response to reviewer comments**

The manuscript ID is PONE-D-21-30169.

Title: Efficient open recruitment and perspectives of host families on medical student homestays in rural Japan

Reviewers’ Comments:

Response to Reviewers’ Questions

**Comments to the Author**

1. If the authors have adequately addressed your comments raised in a previous round of review and you feel that this manuscript is now acceptable for publication, you may indicate that here to bypass the "Comments to the Author" section, enter your conflict of interest statement in the "Confidential to Editor" section, and submit your "Accept" recommendation.

Reviewer #1: All comments have been addressed

Response: Thank you for your positive feedback.

Reviewer #2: (No Response)

Response: Thank you for your positive feedback.

Reviewer #3: All comments have been addressed

Response: Thank you for your positive feedback.

2. Is the manuscript technically sound, and do the data support the conclusions?

The manuscript must describe a technically sound piece of scientific research with data that supports the conclusions.

Reviewer #1: Yes

Response: Thank you for your feedback.

Reviewer #2: Yes

Response: Thank you for your feedback.

Reviewer #3: Yes

Response: Thank you for your feedback.

3. Has the statistical analysis been performed appropriately and rigorously?

Reviewer #1: Yes

Response: Thank you for your feedback.

Reviewer #2: Yes

Response: Thank you for your feedback.

Reviewer #3: Yes

Response: Thank you for your feedback.

4. Have the authors made all data underlying the findings in their manuscript fully available?

The PLOS Data policy requires authors to make all data underlying the findings described in their manuscript fully available without restriction, with rare exception (please refer to the Data Availability Statement in the manuscript PDF file).

The data should be provided as part of the manuscript or its supporting information, or deposited to a public repository.

For example, in addition to summary statistics, the data points behind means, medians and variance measures should be available.

If there are restrictions on publicly sharing data?e.g. participant privacy or use of data from a third party? those must be specified.

Reviewer #1: Yes

Response: Thank you for your feedback.

Reviewer #2: Yes

Response: Thank you for your feedback.

Reviewer #3: Yes

Response: Thank you for your feedback.

5. Is the manuscript presented in an intelligible fashion and written in standard English?

PLOS ONE does not copyedit accepted manuscripts, so the language in submitted articles must be clear, correct, and unambiguous.

Any typographical or grammatical errors should be corrected at revision, so please note any specific errors here.

Reviewer #1: Yes

Response: Thank you for your feedback.

Reviewer #2: Yes

Response: Thank you for your feedback.

Reviewer #3: Yes

Response: Thank you for your feedback.

6. Review Comments to the Author

Please use the space provided to explain your answers to the questions above. You may also include additional comments for the author, including concerns about dual publication, research ethics, or publication ethics. (Please upload your review as an attachment if it exceeds 20,000 characters)

Reviewer #1: The revised manuscript is much clearer and stronger with the revision made, including describing the students who participated in the program (pages 18-19, lines 126-147), expectation of students to interview their host families about their daily lives and health, and group discussions about the medical issues (page 19, lines 155-158). With the statistics of fewer physicians in the rural areas, it would be interesting to see in future studies by the authors about how many (if any) of the student participants in this program (1) volunteer to participate again but in another rural area and/or (2) continue to serve in these communities that they experienced the homestay after they graduate.

Response: We are very grateful for your valuable peer review and for the mention of future studies. The following comments have also been corrected.

Please see below some additional minor recommended suggestions.

Comments on outline of program:

(1) Page 15, line 52 – “Till date, some…” may be clearer if written as ‘To date, some Japanese medical…”; same suggestion for line 55 to use “to date…” instead of “till date…”

Response: Thank you for your comment. We have changed “till date” to “to date” in the text (Page 3, Lines 50, 53–54).

(2) Page 18, line 119 – rather than “I,” suggest using “one of the authors” or “a senior author” sought their assurance in order to maintain consistency in third-person grammar usage

Response: Thank you for your comment. We have changed “I” to “one of the authors” (Page 6, Line 117).

(3) Page 18, lines 134-135 – please clarify, “The students were assigned regardless of their wishes,…” to what is this regarding – which host family to stay with or which area of Hyogo prefecture or some other request by students? Or, is this phrase referring to whether a student is a regional quota student or a non-regional quota student, suggesting that all students have the opportunity to participate?

Response: Thank you for your comment. We have changed this to “The students (regional quota students or non-regional quota students) were assigned randomly and, regardless of their wishes about which host family to stay with or which area of Hyogo prefecture preferences.” (Page 6, Lines 133–135).

(4) Page 19, lines 139-140 – if the program was voluntary, please explain the sentence about students in the Tamba area program who “took part in the homestay program without any particular desire to participate in it.” This seems contradictory that “participation in the community-based medical education program was voluntary…” (page 18, lines 131-132)

Response: Thank you for your comment. This has accordingly been changed to “Their participation in the community-based medical education program was voluntary; however, regardless of their preference for a homestay program, all students assigned to the Tamba area program were required to partake in the homestay program.” (Page 7, Lines 139–142).

(5) Page 19, line 150 – the sentence about bathing and sleeping at the homestay can be revised to state that the students stayed overnight at their host families’ residence.

Response: Thank you for your comment. We have changed this to “The medical students stayed overnight at their host families’ residence.” (Page 7, Line 152).

Overall body of manuscript – please review one more time the sentence structure such as extra periods, etc.

Response: Thank you for your comment.

We have removed an unnecessary period (Page 9, Line 193).

We added a space between “…Spain, and” and “the United…” (Page 15, Line 297).

We pluralized “healthcare professionals” (Page 17, Line 325).

We reviewed the text again and hired a professional editing company to ensure the language is professional.

Reviewer #2: Thank you for the revisions of this manuscript. Listed below are requests for clarification, grammatical suggestions and comments.

Title:
• Line 1: I believe family should be pleural – “Efficient open recruitment and perspectives of host families on medical student homestays in rural Japan”

Response: Thank you for your comment. We have changed this to “Efficient open recruitment and perspectives of host families on medical student homestays in rural Japan” (Page 1, Line 1).

Introduction:

• Lines 52 and 55-56: It should be “To date” instead of “Till date”

Response: Thank you for your comment. We have changed “till date” to “to date” in the text (Page 3, Lines 50, 53–54).

Materials and Methods:

• Lines 123-125: Please clarify the sentence, “During the annual open recruitment period, we request a host family directly from a family who has experienced a host family once”. Do you mean that you ask families who have experienced being a host family in the past? Or do you mean that you ask families whose children were students who experienced a host family? A student would experience a host family, while the family would experience being a host family.

Response: Thank you for your comment. We have changed this to “once each year, we directly requested families who had been hosts in the past, to do so once again.” (Page 6, Lines 122–123).

• Lines 127-128: Please clarify the sentence, “These students were entitled to study funds from Hyogo prefecture after six years of their enrollment, and were obliged to work in the rural area of Hyogo prefecture for 9 years after becoming a doctor in order to be exempted from repaying the funds”. Part of my confusion may be the different structure of medical school, but I believe that medical school is 6 years long in Japan. It sounds like students received study funds after they were in school for six years – so they paid tuition each year and when they graduated, then they received study funds – amount not specified. What are study funds used for? For the nine years of service, does residency count/is there a residency program, or is this after residency?

Response: Thank you for your comment. We changed this to “These students had received scholarships from Hyogo prefecture, were medical students in their sixth year enrollment, and were obliged to work in the rural area of Hyogo prefecture for 9 years after becoming doctors, to be exempted from repaying the scholarship.” (Page 6, Lines 126–128).

• Lines 134-135: Perhaps instead of saying students were assigned “regardless of their wishes”, it could say students were assigned randomly?

Response: Thank you for your comment. We have changed this to “The students (regional or non-regional quota students) were assigned randomly, regardless of their host family or area of Hyogo prefecture preferences” (Page 6, Lines 133–135).

• Lines 139-140: Perhaps instead of saying “without any particular desire to participate in it”, it could say “All the students assigned to the Tamba area program were required to take part in the homestay program”.

Response: Thank you for your comment. We have changed this to “Their participation in the community-based medical education program was voluntary; however, regardless of their preference for a homestay program, all students assigned to the Tamba area program were required to partake in the homestay program” (Page 7, Lines 139–142).

• Lines 146-147: when you use the word “considered” it gives the impression that you thought about it but did it anyway. If the student who was allergic to cats was not placed in a family with cats, it could say, “Only one of the participants was allergic to cats and they were placed in a host family without cats.”

Response: Thank you for your comment. We have changed this to “One participant, who was allergic to cats, was placed in a host family without cats.” (Page 7, Lines 148–149).

• Line 189: There is a period after the word “collected” that should be removed

Response: Thank you for your comment. We have removed the period (Page 9, Line 193).

Discussion:

• Line 299: A space appears to be missing between “…Spain, and” and “the United…”

Response: Thank you for the comment. We have added a space between “…Spain, and” and “the United…” (Page 15, Line 297).

• Line 315: Should be plural - “healthcare professionals”

Response: Thank you for your comment. We have pluralized this to “healthcare professionals” (Page 17, Line 325).

• Reviewer #2, Discussion, second bullet point: The other paper referred to by this reviewer was the article published by the same authors about students (reference 6 – Kenzaka et al, 2020). There were conversations topics reported in this article from the student perspective – was there a difference between the student and host point of view, and if so, possible reasons why?

Response: Thank you for your comment. We have added the following: Regarding the conversation topics that occurred during the homestay, answers of the host families and the students differed, even though they were referring to the same experience. The students’ responses [6] included more health consultations (28.2%) than did the host families’ responses (12.1%). However, expectations of medical students (host family—75.8%, students—61.5% [6]) and expectations from hospitals (host family—69.7%, students—51.2% [6]) were higher in the host family. The topics of conversations are subjective, and it is highly likely that the respondents answered based on their memories. Students may have perceived some conversations as referring to health consultations, even if the host family did not intend it. Furthermore, the host family’s expectations of medical students and from hospitals may not have been fully communicated to the students, even if the former strongly desire communicating the expectation.” (Page 16, Lines 302–312).

Reviewer #3: I have reviewed the article. The authors have addressed all the points and it is worth to publish. This is a very important topic and good way to implement community based teaching.

Response: We are very grateful for your valuable peer review.

6. PLOS authors have the option to publish the peer review history of their article ([what does this mean?](https://journals.plos.org/plosone/s/editorial-and-peer-review-process%23loc-peer-review-history)). If published, this will include your full peer review and any attached files.

If you choose “no”, your identity will remain anonymous but your review may still be made public.

Response: We choose “no.”

**Do you want your identity to be public for this peer review?** For information about this choice, including consent withdrawal, please see our [Privacy Policy](https://www.plos.org/privacy-policy).

Reviewer #1: No

Reviewer #2: No

Reviewer #3: No
